# Impact of the day of the week on the discontinuation of broad-spectrum antibiotic prescriptions; a multi-centered observational study

**DOI:** 10.1038/s41598-021-00206-9

**Published:** 2021-10-21

**Authors:** Hiroyuki Honda, Hideharu Hagiya, Tsukasa Higashionna, Yuto Haruki, Mai Haruki, Shiho Kajita, Kengo Mukuda, Yuji Yokoyama, Yasuhiro Nakano, Hiroko Ogawa, Yasuyo Morimoto, Yoshihisa Hanayama, Setsuko Kanda, Hitomi Kataoka, Hitomi Muguruma, Fumio Otsuka

**Affiliations:** 1 Department of General Medicine, Dentistry and Pharmaceutical Sciences, Okayama University Graduate School of Medicine, 2-5-1 Shikata-cho, Kitaku, Okayama 700-8558 Japan; 2 Department of Pharmacy, Okayama University Hospital, 2-5-1 Shikata-cho, Kitaku, Okayama 700-8558 Japan; 3 Department of Pharmacy, Tsuyama Chuo Hospital, 1756 Kawasaki, Tsuyama, Okayama 708-0841 Japan; 4 Department of Pharmacy, Okayama City Hospital, 3-20-1 Kitanagaseomotemachi, Kitaku, Okayama 700-8557 Japan; 5 Department of Internal Medicine, Yonago Medical Center, 4-17-1 Kuzumo, Yonago, Tottori 863-0006 Japan; 6 Department of Pharmacy, Marugame Medical Center, 219 Tsunomori-cho, Marugame, Kagawa 763-8507 Japan; 7 Department of Pharmacy, Kasaoka City Hospital, 5628-1 Kasaoka, Kasaoka, Okayama 714-0081 Japan; 8 Department of Nursing, Okayama Kyokuto Hospital, 567-1 Kurata, Nakaku, Okayama 703-8265 Japan; 9 Department of Pharymacy, Okayama Memorial Hospital, 7-22 Seikihonmachi, Kitaku, Okayama 700-0862 Japan

**Keywords:** Infectious diseases, Drug regulation

## Abstract

To encourage and guide antimicrobial stewardship team (AST) activity and promote appropriate antibiotic use, we studied the impact of day of the week on the initiation and discontinuation of antibiotic administration. This was a multicenter observational study conducted at 8 Japanese hospitals from April 1 to September 30, 2019, targeting patients who underwent treatment with broad-spectrum antibiotics, such as anti-methicillin-resistant *Staphylococcus aureus* agents and anti-pseudomonal agents. We compared the weekly numbers of initiations and discontinuations of antibiotic prescription on each day of the week or on the days after a holiday. There was no statistical difference in the number of antibiotic initiations on both weekdays and the day after a holiday. However, antibiotic discontinuation was significantly higher from Tuesday onward than Monday and from the second day than the first day after a holiday. Similar trends were observed regardless of the categories of antibiotics, hospital and admission ward, and AST activity. This study suggests that broad-spectrum antibiotics tend to be continued during weekends and holidays and are most likely to be discontinued on Tuesday or the second day after a holiday. This was probably due to behavioral factors beyond medical indications, requiring further antimicrobial stewardship efforts in the future.

## Introduction

One of the most pressing concerns for global public health is antimicrobial resistance (AMR) undermining our healthcare system. The emergence of highly drug-resistant bacteria has been attributed to the excessive and inappropriate use of existing antibiotics^1^, and the rational use of antibiotics is the most important strategy to combat the rapidly escalating threat of AMR. Following the approval of the AMR Global Action Plan in May 2015 by the World Health Organization^2^, we were strongly urged to promote our antimicrobial stewardship program. In Japan, the National Action Plan on AMR was developed in April 2016, raising the issue of AMR to be tackled by our society as a whole^3^.

In general, hospital functionality is lower on weekends than on weekdays because of fewer medical and laboratory staff available. There are several reports on the association between day of the week and health service outcomes. For example, mortality rates for patients electively admitted on weekends may be worse, specifically in surgery, internal medicine, and genecology and obstetrics^4^. The management of and mortality due to myocardial infarction and cerebral stroke is worse among patients admitted on weekends than among those admitted on weekdays^5,6^. In patients hospitalized on weekends for sepsis, in-hospital mortality, admission to the intensive care unit (ICU), and intubation can be worse than in those admitted on weekdays^7^. With regard to antibiotic prescription, inappropriate or excessive use may increase over the weekend in emergency centers^8^ and in primary care settings^9–11^, which cannot simply be explained by the prevalence or incidence of disease. Social or situational factors, such as limited staff, rather than simply medical indications, may have influenced the antibiotic prescription behavior observed in these studies. In a single-center observational study, the in-hospital use of broad-spectrum antibiotics (anti-methicillin-resistant *Staphylococcus aureus* [anti-MRSA] agents, carbapenems, and piperacillin/tazobactam [PIPC/TAZ]) over weekends and holidays was found to be more than was necessary, which may have been due to behavioral factors beyond medical indications^12^.

In this study, we aimed to identify the differences of initiation and discontinuation in the use of broad-spectrum antibiotics between weekdays and weekends in multiple medical institutions.

## Results

**Figure 1:**
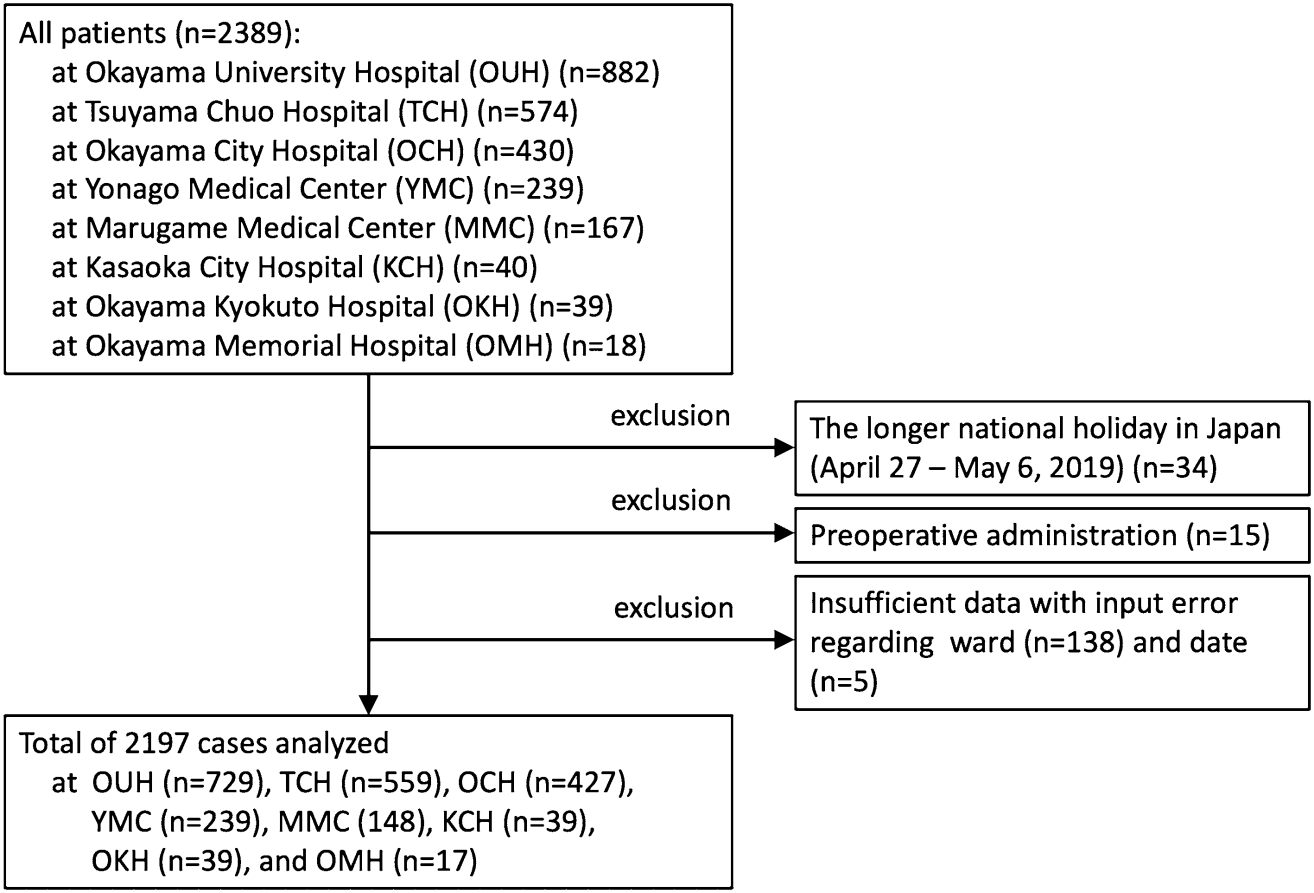
Selection of the study patients.

The selection of the study patients is shown in Fig. 1. Data for all 2389 patients were obtained from OUH (n = 882), TCH (n = 574), OCH (n = 430), YMC (n = 239), MMC (n = 167), KCH (n = 40), OKH (n = 39), and OMH (n = 18). Longer national holidays in Japan (from April 27 to May 6, 2019) were excluded because the antibiotic prescription patterns could have differed over these periods compared with regular weekdays and weekends (n = 34). Preoperative administration of antibiotics was also excluded because the antibiotic prescription patterns could have differed from usual use in infectious diseases (n = 15). Insufficient data with input error regarding ward (n = 138) and date (n = 5) were excluded. Data for the remaining 2197 patients were analyzed.

During the study period, scheduled and unscheduled admissions numbered 714 (32.5%) and 1483 (67.5%), respectively. Almost all the scheduled admissions occurred during weekdays (654, 91.6%), and there were fewer cases of admissions during weekends (60, 8.4%). In addition, unscheduled admissions were more frequent on weekdays (1164, 78.5%) than on weekends (319, 21.5%). There were 1889 (86.0%) patients in the general wards and 308 (14.0%) patients in the ICUs. The number of patients newly administered with anti-MRSA drugs, carbapenems, and PIPC/TAZ were 538 (24.5%), 785 (35.7%), and 874 (39.8%), respectively. The total numbers of days of administration were 5376 days for anti-MRSA drugs, 6186 days for carbapenems, and 7101 days for PIPC/TAZ.

**Figure 2:**
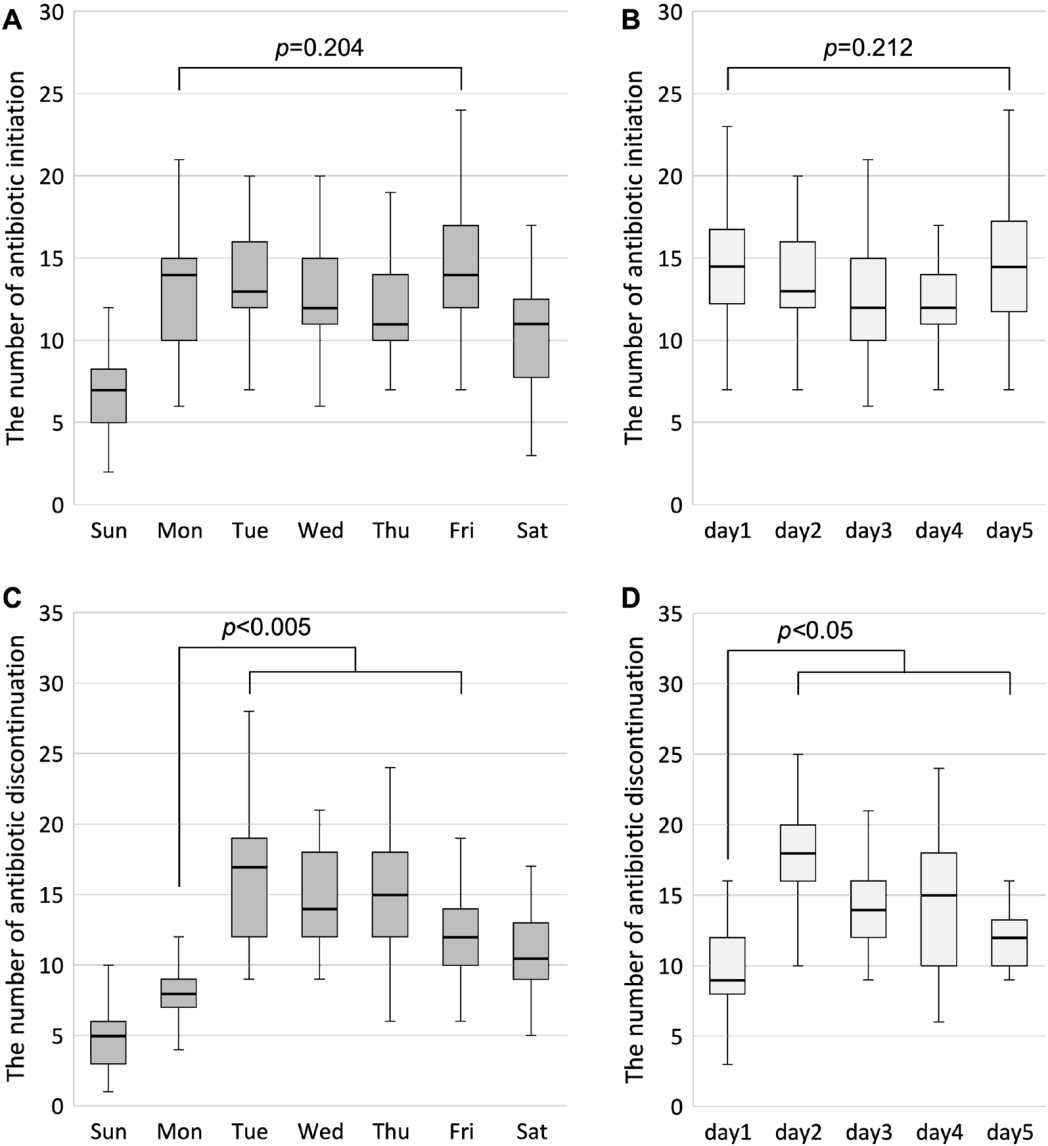
The number of antibiotic initiations and discontinuations on each day of the week (**A**, **C**) and on the day after a holiday (**B**, **D**), overall data. Weekly data are shown as median and box (interquartile range) and whisker (minimum and maximum within 1.5 times the interquartile range) plots. The Kruskal–Wallis test was used to determine the differences among the weekdays (**A**, **C**) and days 1 to 5 (**B**, **D**). Significant differences were observed, and then the Mann–Whitney U test with Bonferroni correction was used to determine the differences in antibiotic discontinuation between days.

The numbers of broad-spectrum antibiotic initiations on each day of the week and on the day after the holiday are shown in Fig. 2A and B. Over a weekly period, there were fewer antibiotic initiations on Saturdays and Sundays than on weekdays. However, there were no significant differences in antibiotic initiations among weekdays (Fig. 2A, *p* = 0.204) and on the day after a holiday (Fig. 2B,  *p* = 0.212).

The numbers of broad-spectrum antibiotic discontinuations on each day of the week and on the days after a holiday are demonstrated in Fig. 2C and D. There were clearly fewer discontinuations on Sundays. The broad-spectrum antibiotics were disproportionately discontinued after Tuesday compared to Monday; *p*-value was < 0.001 between Monday and Tuesday, between Monday and Wednesday, and between Monday and Thursday, and *p-*value was < 0.005 between Monday and Friday (Fig. 2C). Furthermore, the number of discontinuations was significantly higher after the second day following a holiday; *p-*value was < 0.001 between days 1 and 2, between days 1 and 3, and between days 2 and 5, *p-*value was < 0.01 between days 1 and 4, and *p*-value was < 0.05 between days 1 and 5 and between days 2 and 3 (Fig. 2D).

**Figure 3:**
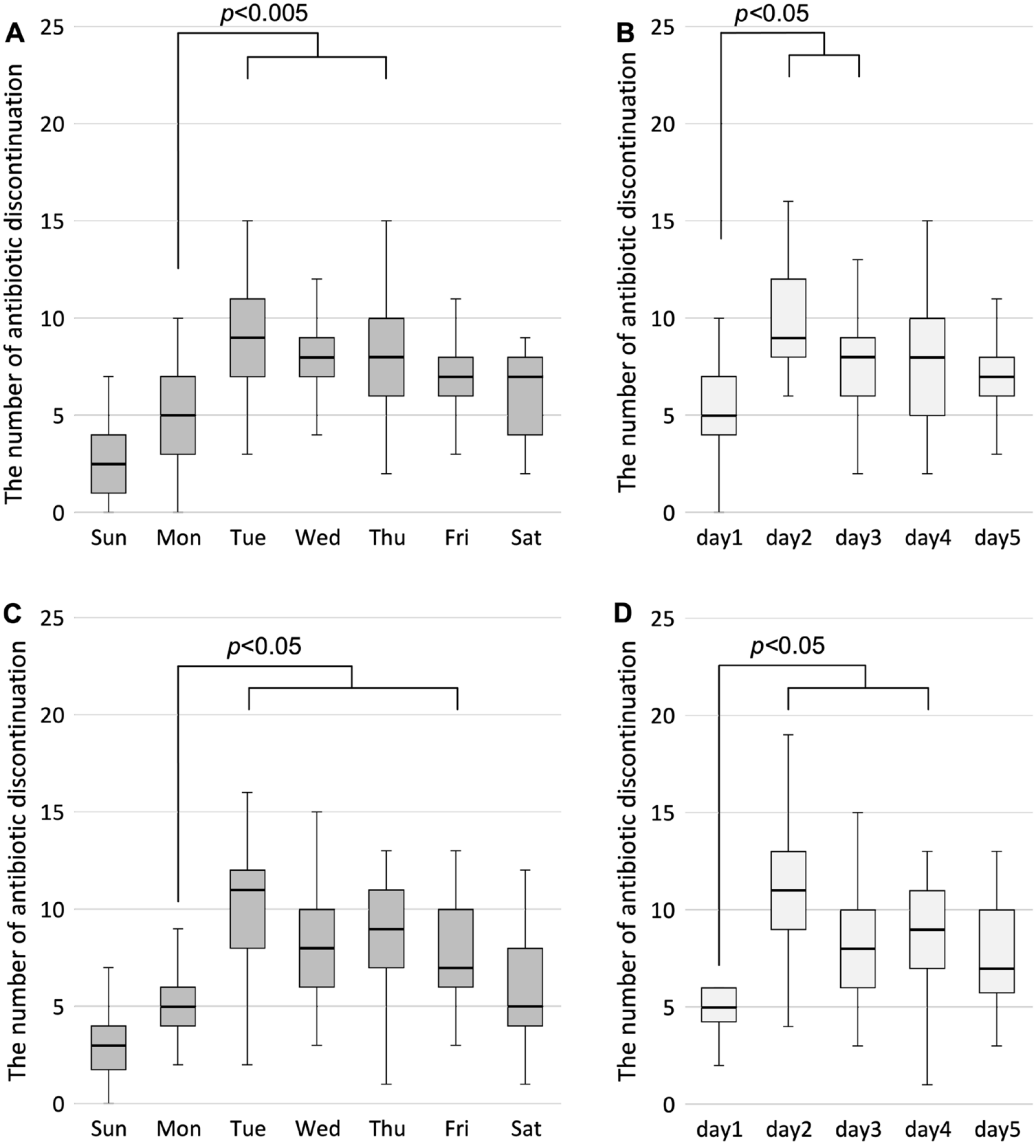
The number of antibiotic discontinuations on each day of the week and on the day after a holiday among 2 hospitals with antimicrobial stewardship team activities (**A**, **B**) and among 2 hospitals where infectious disease specialists work full-time (**C**, **D**). Weekly data are shown as median and box (interquartile range) and whisker (minimum and maximum within 1.5 times the interquartile range) plots. The Kruskal–Wallis test was used to determine the differences among the weekdays (**A**, **C**) and days 1 to 5 (**B**, **D**). Significant differences were observed, and then the Mann–Whitney U test with Bonferroni correction was used to determine the differences in antibiotic discontinuation between days. AST activities include direct advice on antibiotic prescription to the attending physician at Tsuyama Chuo Hospital (TCH) and at Okayama City Hospital. Infectious disease specialists work full-time, and attending doctors can consult them about antibiotic prescription at Okayama University Hospital and TCH.

A similar pattern was found in a sub-analysis with data in TCH and OCH with AST activity, including direct advice on antibiotic prescription to attending physicians (Fig. 3A and B). Broad-spectrum antibiotics were disproportionately discontinued after Tuesday compared to Monday; *p* value was < 0.001 between Monday and Tuesday, and *p*-value was < 0.005 between Monday and Wednesday and between Monday and Thursday (Fig. 3A) and after the second day following a holiday. Moreover, *p-*value was < 0.001 between days 1 and 2, and *p-*value was < 0.05 between days 1 and 3 (Fig. 3B). A similar trend was observed in the data obtained from OUH and TCH, where infectious disease specialists work full-time and attending doctors can consult them about antibiotic prescription. The number of discontinuations was significantly higher after Tuesday than after Monday. Additionally, *p-*value was < 0.001 between Monday and Tuesday, *p-*value was < 0.005 between Monday and Wednesday and between Monday and Thursday, and *p-*value was < 0.05 between Monday and Friday (Fig. 3C) and after the second day following a holiday. Furthermore, *p*-value was < 0.001 between days 1 and 2, *p*-value was < 0.01 between days 1 and 4, and *p-*value was < 0.05 between days 1 and 3 (Fig. 3D). As shown in Table 1, sub-analyses in the types of antibiotics, hospitals, and ward categories also showed that antibiotics were more significantly discontinued on Tuesday than on Monday (*p* < 0.01), or on the second day than on the first day after the holiday (*p* < 0.05). In the ICUs, there were no significant differences among the weekdays, but there was a significant difference between the first day and second day after a holiday (*p* < 0.05). We performed an analysis of the relationship between the day of the week and the duration of treatment. There was no significant difference in the duration of treatment depending on the day of the week or the day after the holiday (Supplement 1).

**Table 1:** The number of antibiotic discontinuation on weekday (A) and on the day after holiday (B), by antibiotic, hospital and ward categories.

A. Weekday	Mon		Tus		Wed		Thu		Fri		*p*-value^a^	*p*-value^b^
												(Mon vs Tus)
**Antibiotics**												
Anti-MRSA drugs	1 [1, 3]		4 [2, 5]		4 [2, 5]		3 [2, 5]		3 [2, 4]		***p < 0.001	***p < 0.001
Anti-pseudomonal drugs	7 [5, 8]		14 [10, 15]		11 [10, 13]		11 [8, 13]		9 [8, 11]		***p < 0.001	***p < 0.001
**Hospital**												
University hospital	3 [2, 4]		6 [4, 8]		4 [3, 6]		5 [4, 6]		4 [2, 6]		**p < 0.01	***p < 0.001
City hospital	5 [4, 7]		11 [8, 13]		9 [8, 12]		11 [8, 13]		8 [7, 9]		***p < 0.001	***p < 0.001
**Ward**												
ICU	1 [0, 2]		1 [1, 3]		2 [1, 3]		2 [2, 3]		2 [1, 3]		0.0717	–
General ward	8 [6, 9]		16 [11, 17]		13 [11, 16]		13 [9, 15]		10 [9, 12]		***p < 0.001	***p < 0.001

B. The day after holiday	Day 1		Day 2		Day 3		Day 4		Day 5		*p*-value^a^	*p*-value^b^
												(Day 1 vs day 2)
**Antibiotics**												
Anti-MRSA drugs	2 [1, 3]		5 [3, 6]		3 [2, 4]		3 [2, 5]		3 [2, 5]		***p < 0.001	***p < 0.001
Anti-pseudomonal drugs	8 [6, 9]		4 [11, 16]		11 [9, 13]		10 [8, 13]		9 [8, 11]		***p < 0.001	***p < 0.001
**Hospital**												
University hospital	3 [2, 4]		7 [4, 9]		4 [3, 6]		5 [3, 6]		4 [2, 5]		***p < 0.001	***p < 0.001
City hospital	6 [5, 8]		12 [9, 14]		9 [8, 12]		10 [6, 13]		8 [7, 9]		***p < 0.001	***p < 0.001
**Ward**												
ICU	1 [0, 2]		2 [1, 4]		1 [1, 2]		2 [1, 3]		2 [1, 3]		*p < 0.05	*p < 0.05
General ward	8 [6, 11]		16 [13, 18]		13 [10, 14]		13 [8, 14]		10 [9, 11]		***p < 0.001	***p < 0.001

Values are shown as median [interquartile range] and were statistically analyzed by ^a^the Kruskal–Wallis test to determine differences among the weekdays (A) and day1 to day 5 (B). Significant differences were observed, and then ^b^the Mann–Whitney U test with Bonferroni correction was used to determine differences in discontinuation between Monday and Tuesday (A), and between day1 and day2 (B). Significant level was set at *p* < 0.05. MRSA: methicillin-resistant *Staphylococcus aureus*, ICU: intensive care unit. Anti-MRSA drugs include vancomycin, teicoplanin, linezolid, daptomycin, and arbekacin, and anti-Pseudomonal drugs include carbapenems (meropenem, imipenem, doripenem, and biapenem) and piperacillin/tazobactam.

## Discussion

The study revealed that the number of broad-spectrum antibiotic discontinuations was uneven throughout the week, specifically on weekdays, with significantly more antibiotics discontinued on Tuesday or later than on Monday, and on the second day or later than on the first day after the holiday. This trend was also observed in hospitals with AST activity and a direct consultation with infectious disease specialists on antimicrobial prescriptions. In addition, this pattern was also true even when stratifying the data into types of antimicrobial agents (anti-MRSA agents and anti-pseudomonal agents), hospitals (university hospital and regional hospital), and ward categories (general ward and ICU). And there was no difference between the day of the week and the duration of treatment. The onset of infectious disease is not biased toward any particular day; therefore, the termination of antibiotic administration should be evenly distributed over the week. However, our results showed a particular variation within a week, suggesting that there are other factors apart from medical indications. Multiple factors, such as staffing shortages, may influence antibiotic prescription, as shown in previous reports^8–12^.

There are several possible explanations for the tendency of antibiotic discontinuation. A single-center observational study at Osaka University Hospital, Japan, reported significantly more discontinuation of antibiotics on Tuesday or the second day after a holiday, similar to the results of the present study. The AST at the hospital reviewed broad-spectrum antibiotics prescribed on a daily basis throughout the weekdays, but there was an uneven discontinuation of antibiotics. In academic medical institutions, it takes a full day for young clinicians to review laboratory data, discuss laboratory results with their attending physicians, and finally decide to discontinue or change antibiotic treatments. This may be one of the reasons for the discontinuation of antibiotics throughout the week^12^. Sub-analysis for each of the university and community hospitals in this study found similar patterns. In regional hospitals with few clinicians, they may be busy on weekdays with outpatient duties, which may delay the decision on the treatment plan for inpatients. Although AST interventions were effective in terms of appropriate use of antibiotics^13,14^, the results were similar even when AST activities and consultation with specialists were available. The fact that ASTs and specialists are generally working only on weekdays may have influenced the present study. The relatively increased number of antibiotic discontinuations on weekdays after Tuesday and after the second day of the holiday may also have been attributed to AST activity that starts up on Monday or the first day of the week.

Solutions to disproportionate antibiotic prescription should be discussed. Recent British literature on hospital quality improvement reports that it would be valuable to conduct a review of antibiotic administration before the weekend^15^. Up-to-date notes on treatment left for the weekend staff can reduce their burden and make physicians responsible for decision-making. By assessing patients properly on a daily basis, we believe that antibiotic use can be further optimized. The decision to prescribe antibiotics is also influenced by the physician’s background, including their medical careers and specialty^16^. These personal trends in antibiotic prescription should also be well managed by the activities of the AST^13,14^. Additional hospital staffing during the holidays could improve antibiotic use and would be well worth the additional expenses. A hospitalist system could be a solution, as it would allow staff to work shifts and focus solely on ward duties, regardless of weekdays and holidays^17^.

This study has several limitations. First, the study did not analyze patient outcomes, which are important in assessing the effectiveness of AST activity. Second, it did not analyze the organs that are infected. The diagnosis of infection can also affect the duration and timing of antibiotic initiation and discontinuation. Third, attending physicians prescribing antibiotics have not been analyzed. The specialty and experience of medical doctors can influence the pattern of antibiotic prescription.

In summary, the study found a bias in discontinuing broad-spectrum antibiotics throughout the week, regardless of the types of facilities, wards, and AST activities. This result is expected to encourage and guide AST activity, leading to the appropriate use of antibiotics in this age of growing AMR. In particular, with regard to the use of broad-spectrum antibiotics, AST activity is expected even on weekends and holidays. Modifying daily AST activities, such as directing and scheduling the use of antibiotics prior to weekends, is needed to ensure that ward staff are able to cope with weekends and holidays when staffing is short. Differences in staffing between weekdays and weekends are common worldwide; thus, similar phenomena to those reported in this study may be observed elsewhere. Further research in different medical cultures to confirm the generalizability of the results observed in this study will be needed.

## Methods

We conducted a multicenter retrospective observational study on the in-hospital use of broad-spectrum antibiotics (defined below) at Okayama University Hospital (OUH) and 7 community hospitals. Two of these hospitals (Marugame Medical Center [MMC] and Kasaoka City Hospital [KCH]) were associated with the endowed course of Okayama University Graduate School of Medicine, Dentistry, and Pharmaceutical Sciences, which conducts clinical research and social implementation trials for the education of general practitioners focusing on regional characteristics in the Setouchi Marine Area. Other facilities included Tsuyama Chuo Hospital (TCH), Okayama City Hospital (OCH), Yonago Medical Center (YMC), Okayama Kyokuto Hospital (OKH), and Okayama Memorial Hospital (OMH). These hospitals do not require pre-prescription authorization to initiate antibiotic treatment, and both attending physicians and residents can process orders for antibiotics. At OUH and TCH, infectious disease specialists work full-time, and they are available to discuss antimicrobial prescription with the attending physicians. At OUH, TCH, OCH, YMC, and OKH, the infection control team conducts prospective audits and feedback as an AST activity on weekdays, but not on weekends. This feedback is provided in the medical record alone, but at TCH and OCH, the attending physicians are contacted directly by AST members if necessary.

The study period was from April 1 to September 30, 2019 (6 months). A collection of public holidays on the Japanese calendar (from April 27 to May 6, 2019) was excluded from the study because during this period, antibiotic prescription patterns were different from those on regular weekdays and weekends. All hospitalized patients who received broad-spectrum antibiotics during this period were included in the study. The data were obtained from the electronic medical records. The information about enrollment in the study was disclosed on each hospital’s website and by poster notice at each hospital with an opportunity for a denial of participation, and we provided a contact point for participants’ opt-out. A need of informed consent from each participant was waived and the study protocol was approved by the Ethical Committees of OUH (No. 2001-013) and adhered to the tenets of the Declaration of Helsinki and the Ethical Guidelines for Medical and Health Research Involving Human Subjects.

Broad-spectrum antibiotics were defined as follows: (i) anti-MRSA drugs, including vancomycin, teicoplanin, linezolid, daptomycin, and arbekacin, and (ii) anti-pseudomonal drugs, such as carbapenems (meropenem, imipenem/cilastatin, doripenem, and biapenem) and PIPC/TAZ.

The weekdays were defined as Monday to Friday and weekends as Saturday and Sunday. Holidays were defined as national days off. Routine care was usually provided on weekdays. Hospital staff functionality, both numerically and qualitatively, was reduced during weekends and holidays, but not during weekdays. In general wards, there are usually no patient handoffs among attending doctors. We analyzed the initiation and discontinuation of antibiotic prescription on each day of the week. In addition, we investigated these patterns on the days after holidays to evaluate the influence of holidays on antibiotic prescription.

For treatment initiation, we compared the weekly numbers of antibiotic prescriptions that started on each day of the week. For treatment discontinuation, we compared the weekly numbers of antibiotic prescriptions discontinued on each day of the week or on the days after a holiday. We also performed sub-analysis individually between anti-MRSA and anti-pseudomonal drugs, between the university hospital (OUH) and the other regional hospitals, between general wards and ICUs, two hospitals with AST activity (OCH and TCH), and two hospitals with full-time infectious disease specialists (OUH and TCH). For the comparison of continuous variables, we applied the Kruskal–Wallis test and the Mann–Whitney U test with Bonferroni correction as a *post-hoc* procedure. Analyses were performed using the EZR, version 3.5.2, which is a graphical user interface for R (The R Foundation for Statistical Computing, Vienna, Austria)^18^. Statistical significance was set at a *p*-value < 0.05.

## Supplementary Information

Supplementary Information 1.

Supplementary Information 2.

## Abbreviations

AMR: Antimicrobial resistance
AST: Antimicrobial stewardship team
ICU: Intensive care unit
PIPC/TAZ: Piperacillin/tazobactam
MRSA: Methicillin resistant *Staphylococcus aureus*
