# Supplementary figures and images for: Impact of the day of the week on the discontinuation of broad-spectrum antibiotic prescriptions; a multi-centered observational study
Source: Sci Rep. 2021 Oct 21;11:20784. doi: 10.1038/s41598-021-00206-9 (PMC8531020; doi:10.1038/s41598-021-00206-9)

**A**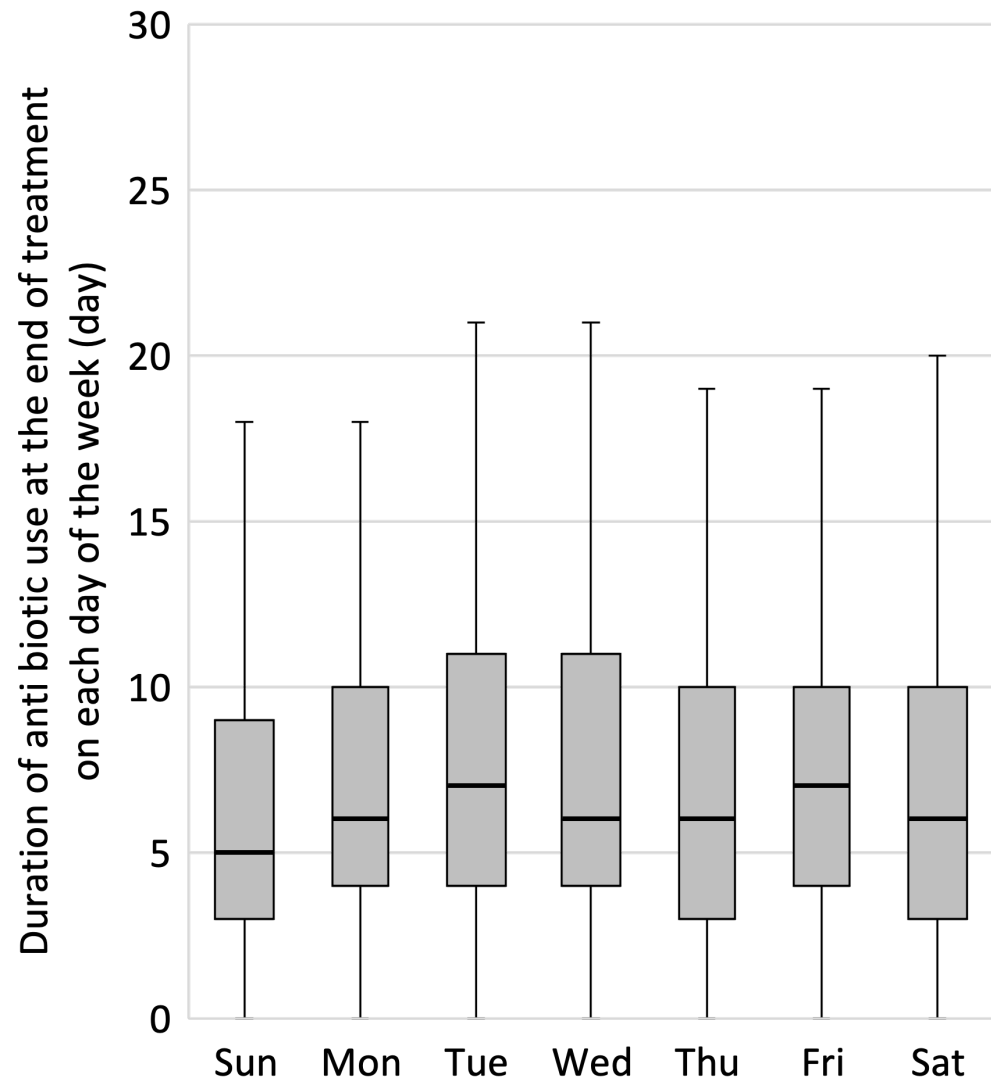**B**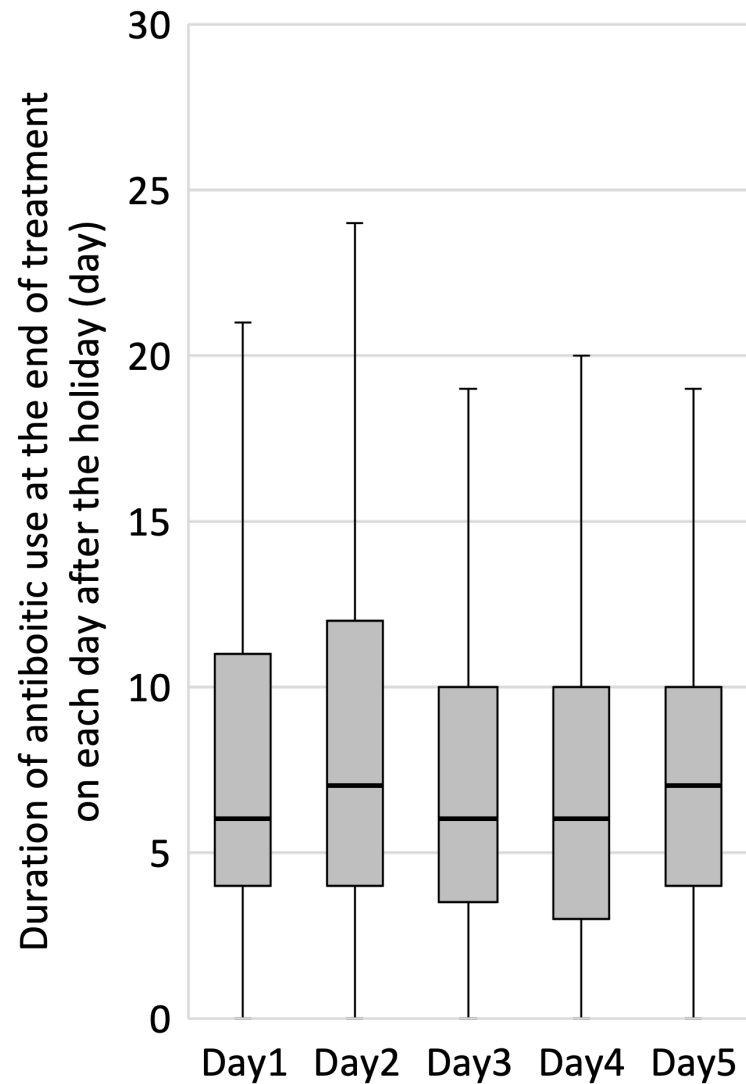

Supplement: Supplementary file 1 — Supplementary Information 1. [file 41598_2021_206_MOESM1_ESM.pdf]
