# Supplementary material for: Impact of the day of the week on the discontinuation of broad-spectrum antibiotic prescriptions; a multi-centered observational study
Source: Sci Rep. 2021 Oct 21;11:20784. doi: 10.1038/s41598-021-00206-9 (PMC8531020; doi:10.1038/s41598-021-00206-9)
Supplement: Supplementary file 2 — Supplementary Information 2. [file 41598_2021_206_MOESM2_ESM.docx]

**Supplement 1. Duration of antibiotic use at the end of treatment on each day of the week (A) and on the day after a holiday (B).**

Weekly data are shown as median and box (interquartile range) and whisker (minimum and maximum within 1.5 times the interquartile range) plots. The Kruskal–Wallis test was used to determine the differences among the weekdays (A) and days 1 to 5 (B).
